# Supplementary material for: Modeling glioblastoma heterogeneity as a dynamic network of cell states
Source: Mol Syst Biol. 2021 Sep 16;17(9):e10105. doi: 10.15252/msb.202010105 (PMC8444284; doi:10.15252/msb.202010105)
Supplement: Supplementary file 5 — Source Data for Figure 3 [file MSB-17-e10105-s001.zip › Figure3A_sourcedata/GSEA_3065/nosigdb_state1.GseaPreranked.1622718409535/gsea_report_for_na_pos_1622718409535.html]

Report for na\_pos 1622718409535 [GSEA]

| GS  follow link to MSigDB | GS DETAILS | SIZE | ES | NES | NOM p-val | FDR q-val | FWER p-val | RANK AT MAX | LEADING EDGE || 1 | LARSSON\_STATE\_1 | Details ... | 99 | 1.00 | 3.61 | 0.000 | 0.000 | 0.000 | 98 | tags=100%, list=1%, signal=100% |
| 2 | NB\_KI\_CLUSTER1 | Details ... | 32 | 0.86 | 2.56 | 0.000 | 0.000 | 0.000 | 811 | tags=84%, list=8%, signal=92% |
| 3 | RICHARDS\_NEFTEL\_CELL\_2019\_MES1 | Details ... | 40 | 0.76 | 2.35 | 0.000 | 0.000 | 0.000 | 230 | tags=43%, list=2%, signal=43% |
| 4 | SUVA\_MES1\_PMID\_31327527 | Details ... | 40 | 0.76 | 2.27 | 0.000 | 0.000 | 0.000 | 230 | tags=43%, list=2%, signal=43% |
| 5 | LARSSON\_STATE\_4 | Details ... | 101 | 0.60 | 2.17 | 0.000 | 0.000 | 0.000 | 1651 | tags=46%, list=17%, signal=54% |
| 6 | NB\_KI\_CLUSTER3 | Details ... | 162 | 0.57 | 2.15 | 0.000 | 0.000 | 0.000 | 1470 | tags=36%, list=15%, signal=42% |
| 7 | DOROSZKO\_U3180MG\_CLUSTER\_3 | Details ... | 474 | 0.50 | 2.14 | 0.000 | 0.000 | 0.000 | 2036 | tags=41%, list=21%, signal=49% |
| 8 | WANG\_MESENCHYMAL\_PMID\_28697342 | Details ... | 29 | 0.74 | 2.13 | 0.000 | 0.000 | 0.000 | 1482 | tags=52%, list=15%, signal=61% |
| 9 | DOROSZKO\_U3180MG\_CLUSTER\_4 | Details ... | 168 | 0.56 | 2.13 | 0.000 | 0.000 | 0.000 | 1842 | tags=47%, list=19%, signal=57% |
| 10 | DALMO\_EXTINCTION\_UNPUB | Details ... | 29 | 0.74 | 2.10 | 0.000 | 0.000 | 0.000 | 1482 | tags=52%, list=15%, signal=61% |
| 11 | RICHARDS\_NOWAKOWSKI\_SCIENCE\_2017\_RG\_EARLY\_UPREG | Details ... | 447 | 0.49 | 2.07 | 0.000 | 0.000 | 0.000 | 1445 | tags=37%, list=15%, signal=42% |
| 12 | DOROSZKO\_U3013MG\_CLUSTER\_2 | Details ... | 210 | 0.50 | 1.98 | 0.000 | 0.000 | 0.004 | 2028 | tags=42%, list=21%, signal=52% |
| 13 | RICHARDS\_VERHAAK\_CANCERCELL\_2010\_MESENCHYMAL | Details ... | 116 | 0.53 | 1.94 | 0.000 | 0.000 | 0.006 | 1845 | tags=39%, list=19%, signal=47% |
| 14 | FELDMAN\_2020\_G1 | Details ... | 152 | 0.50 | 1.92 | 0.000 | 0.000 | 0.007 | 1651 | tags=53%, list=17%, signal=62% |
| 15 | FELDMAN\_2020\_LATEG1 | Details ... | 93 | 0.52 | 1.89 | 0.000 | 0.001 | 0.016 | 1835 | tags=62%, list=19%, signal=76% |
| 16 | RICHARDS\_NEFTEL\_CELL\_2019\_G2\_M | Details ... | 45 | 0.60 | 1.87 | 0.000 | 0.001 | 0.020 | 2566 | tags=62%, list=26%, signal=84% |
| 17 | SUVA\_G2M\_PMID\_31327527 | Details ... | 45 | 0.60 | 1.87 | 0.000 | 0.001 | 0.021 | 2566 | tags=62%, list=26%, signal=84% |
| 18 | NB\_KI\_CLUSTER16 | Details ... | 81 | 0.54 | 1.86 | 0.000 | 0.001 | 0.023 | 1513 | tags=42%, list=15%, signal=49% |
| 19 | FELDMAN\_2020\_S-G2 | Details ... | 334 | 0.44 | 1.82 | 0.000 | 0.002 | 0.035 | 1573 | tags=43%, list=16%, signal=50% |
| 20 | RICHARDS\_NEFTEL\_CELL\_2019\_MES2 | Details ... | 45 | 0.58 | 1.80 | 0.000 | 0.002 | 0.048 | 875 | tags=33%, list=9%, signal=36% |
| 21 | FELDMAN\_2020\_NEURAL G0 |  | 376 | 0.43 | 1.80 | 0.000 | 0.002 | 0.049 | 780 | tags=35%, list=8%, signal=36% |
| 22 | SUVA\_MES2\_PMID\_31327527 |  | 45 | 0.58 | 1.80 | 0.002 | 0.002 | 0.049 | 875 | tags=33%, list=9%, signal=36% |
| 23 | NB\_KI\_CLUSTER11 |  | 52 | 0.57 | 1.79 | 0.000 | 0.002 | 0.052 | 1244 | tags=42%, list=13%, signal=48% |
| 24 | RICHARDS\_VERHAAK\_CANCERCELL\_2010\_NEURAL |  | 62 | 0.53 | 1.78 | 0.002 | 0.002 | 0.055 | 2258 | tags=35%, list=23%, signal=46% |
| 25 | SINGH\_SOX2\_DOWNREGULATED\_TRANSCRIPTS\_PMID\_28122245 |  | 107 | 0.49 | 1.77 | 0.000 | 0.002 | 0.070 | 1031 | tags=25%, list=10%, signal=28% |
| 26 | FELDMAN\_2020\_G2-M |  | 292 | 0.43 | 1.76 | 0.000 | 0.003 | 0.080 | 1484 | tags=44%, list=15%, signal=50% |
| 27 | OLSEN\_ADRENERGIC |  | 66 | 0.52 | 1.76 | 0.002 | 0.003 | 0.080 | 763 | tags=42%, list=8%, signal=46% |
| 28 | OLSEN\_MESENCHYMAL |  | 72 | 0.52 | 1.75 | 0.000 | 0.003 | 0.093 | 753 | tags=38%, list=8%, signal=40% |
| 29 | RICHARDS\_ZHONG\_NATURE\_2018\_NPCS\_UPREG |  | 125 | 0.46 | 1.70 | 0.000 | 0.005 | 0.160 | 1949 | tags=39%, list=20%, signal=48% |
| 30 | NB\_KI\_CLUSTER5 |  | 86 | 0.49 | 1.69 | 0.002 | 0.006 | 0.179 | 1794 | tags=37%, list=18%, signal=45% |
| 31 | IVY\_CTPAN |  | 107 | 0.45 | 1.65 | 0.000 | 0.008 | 0.268 | 922 | tags=25%, list=9%, signal=28% |
| 32 | RICHARDS\_NOWAKOWSKI\_SCIENCE\_2017\_MGE\_RG2\_UPREG |  | 445 | 0.38 | 1.60 | 0.000 | 0.013 | 0.387 | 1570 | tags=34%, list=16%, signal=39% |
| 33 | GANDAL\_AUTISM\_UP\_PMID29439242 |  | 86 | 0.45 | 1.56 | 0.011 | 0.019 | 0.534 | 931 | tags=26%, list=9%, signal=28% |
| 34 | RICHARDS\_LIDDELOW\_NATURE\_2017\_A2\_REACTIVE\_ASTROCYTES |  | 29 | 0.53 | 1.53 | 0.016 | 0.023 | 0.603 | 931 | tags=48%, list=9%, signal=53% |
| 35 | NB\_KI\_CLUSTER18 |  | 73 | 0.44 | 1.50 | 0.025 | 0.027 | 0.682 | 753 | tags=33%, list=8%, signal=35% |
| 36 | FELDMAN\_2020\_S |  | 152 | 0.39 | 1.50 | 0.009 | 0.027 | 0.693 | 1594 | tags=40%, list=16%, signal=47% |
| 37 | PARADA\_T1\_UP\_26461091 |  | 66 | 0.44 | 1.47 | 0.024 | 0.035 | 0.786 | 1155 | tags=32%, list=12%, signal=36% |
| 38 | RICHARDS\_NOWAKOWSKI\_SCIENCE\_2017\_IPC\_NEN3\_UPREG |  | 233 | 0.36 | 1.46 | 0.004 | 0.036 | 0.808 | 855 | tags=24%, list=9%, signal=26% |
| 39 | FELDMAN\_2020\_M-EARLYG1 |  | 112 | 0.40 | 1.44 | 0.006 | 0.040 | 0.844 | 764 | tags=38%, list=8%, signal=41% |
| 40 | DOROSZKO\_U3054MG\_CLUSTER\_6 |  | 117 | 0.39 | 1.42 | 0.022 | 0.046 | 0.884 | 1943 | tags=35%, list=20%, signal=43% |
| 41 | DALMO\_BARCODENUMBER\_UNPUB |  | 195 | 0.35 | 1.37 | 0.015 | 0.065 | 0.949 | 1082 | tags=26%, list=11%, signal=28% |
| 42 | IVY\_LE |  | 31 | 0.45 | 1.31 | 0.126 | 0.105 | 0.992 | 1537 | tags=32%, list=16%, signal=38% |
| 43 | SEURAT\_CELLCYCLE |  | 90 | 0.36 | 1.29 | 0.087 | 0.116 | 0.997 | 2420 | tags=41%, list=24%, signal=54% |
| 44 | NB\_KI\_CLUSTER12 |  | 48 | 0.40 | 1.26 | 0.139 | 0.136 | 0.999 | 2251 | tags=38%, list=23%, signal=48% |
| 45 | DOROSZKO\_U3180MG\_CLUSTER\_6 |  | 41 | 0.41 | 1.26 | 0.139 | 0.133 | 0.999 | 1824 | tags=37%, list=18%, signal=45% |
| 46 | RICHARDS\_NOWAKOWSKI\_SCIENCE\_2017\_IPC\_NEN1\_UPREG\_1 |  | 135 | 0.33 | 1.24 | 0.075 | 0.149 | 1.000 | 798 | tags=21%, list=8%, signal=22% |
| 47 | ANASTASSIOU\_MULTICANCER\_INVASIVENESS\_PMID\_22208948 |  | 33 | 0.42 | 1.24 | 0.157 | 0.147 | 1.000 | 1002 | tags=21%, list=10%, signal=24% |
| 48 | ALMSTEDT\_RISK |  | 31 | 0.42 | 1.23 | 0.171 | 0.157 | 1.000 | 700 | tags=29%, list=7%, signal=31% |
| 49 | GAROFANO\_IAVARONE\_LIST\_OF\_NEURAL\_PROGENITOR\_MARKER\_GENES |  | 85 | 0.35 | 1.22 | 0.115 | 0.157 | 1.000 | 1794 | tags=41%, list=18%, signal=50% |
| 50 | IVY\_CTMVP |  | 60 | 0.36 | 1.19 | 0.167 | 0.190 | 1.000 | 1877 | tags=42%, list=19%, signal=51% |
| 51 | DOROSZKO\_U3180MG\_CLUSTER\_5 |  | 33 | 0.41 | 1.18 | 0.237 | 0.200 | 1.000 | 473 | tags=24%, list=5%, signal=25% |
| 52 | KLING\_GBM\_PHENO\_PMID\_27667176 |  | 149 | 0.31 | 1.18 | 0.115 | 0.198 | 1.000 | 1581 | tags=24%, list=16%, signal=28% |
| 53 | PARADA\_T2\_UP\_26461091 |  | 66 | 0.35 | 1.18 | 0.201 | 0.197 | 1.000 | 1146 | tags=27%, list=12%, signal=31% |
| 54 | NB\_KI\_CLUSTER22 |  | 155 | 0.31 | 1.17 | 0.133 | 0.208 | 1.000 | 1775 | tags=21%, list=18%, signal=26% |
| 55 | RICHARDS\_NOWAKOWSKI\_SCIENCE\_2017\_EN\_PFC3\_UPREG |  | 208 | 0.29 | 1.16 | 0.140 | 0.211 | 1.000 | 1093 | tags=19%, list=11%, signal=21% |
| 56 | GANDAL\_DEPRESSION\_DN\_PMID29439242 |  | 35 | 0.38 | 1.14 | 0.270 | 0.238 | 1.000 | 1225 | tags=26%, list=12%, signal=29% |
| 57 | NB\_KI\_CLUSTER13 |  | 131 | 0.29 | 1.07 | 0.287 | 0.343 | 1.000 | 1468 | tags=18%, list=15%, signal=21% |
| 58 | KRONA\_ROSEN\_PERIVASCULAR\_INVASION |  | 57 | 0.32 | 1.05 | 0.342 | 0.389 | 1.000 | 1409 | tags=18%, list=14%, signal=20% |
| 59 | GANDAL\_BIPOLAR\_DN\_PMID29439242 |  | 18 | 0.41 | 1.03 | 0.428 | 0.425 | 1.000 | 1401 | tags=44%, list=14%, signal=52% |
| 60 | ANGIOGENESIS\_GO\_0001525 |  | 21 | 0.37 | 1.01 | 0.446 | 0.468 | 1.000 | 291 | tags=14%, list=3%, signal=15% |
| 61 | GANDAL\_IBD\_DN\_PMID29439242 |  | 27 | 0.32 | 0.89 | 0.600 | 0.752 | 1.000 | 1573 | tags=30%, list=16%, signal=35% |
| 62 | PARADA\_T1\_DN\_26461091 |  | 51 | 0.28 | 0.88 | 0.689 | 0.766 | 1.000 | 893 | tags=12%, list=9%, signal=13% |
| 63 | RICHARDS\_NEFTEL\_CELL\_2019\_G1\_S |  | 27 | 0.27 | 0.77 | 0.796 | 0.925 | 1.000 | 2567 | tags=30%, list=26%, signal=40% |
| 64 | SUVA\_G1S\_PMID\_31327527 |  | 27 | 0.27 | 0.77 | 0.835 | 0.912 | 1.000 | 2567 | tags=30%, list=26%, signal=40% |
Table: Gene sets enriched in phenotype **na **[plain text format]****
